# Does Surgical Approach Influence Complication Rate of Hip Hemiarthroplasty for Femoral Neck Fractures? A Literature Review and Meta-Analysis

**DOI:** 10.3390/medicina59071220

**Published:** 2023-06-29

**Authors:** Matteo Filippini, Marta Bortoli, Andrea Montanari, Andrea Pace, Lorenzo Di Prinzio, Gianluca Lonardo, Stefania Claudia Parisi, Valentina Persiani, Roberto De Cristofaro, Andrea Sambri, Massimiliano De Paolis, Michele Fiore

**Affiliations:** 1 Alma Mater Studiorum, University of Bologna, 40126 Bologna, Italy; matteo.filippini@ior.it (M.F.); marta.bortoli@ior.it (M.B.); andrea.montanari36@studio.unibo.it (A.M.); andrea.pace@ior.it (A.P.); lorenzo.diprinzio@ior.it (L.D.P.); stefaniaclaudiaparisi@hotmail.it (S.C.P.); michele.fiore@ior.it (M.F.); 2 Orthopedics and Traumatology Department, IRCCS Azienda Ospedaliero-Universitaria di Bologna, 40138 Bologna, Italy; gianluca.lonardo@gmail.com (G.L.); valentina.persiani@aosp.bo.it (V.P.); roberto.decristofaro@aosp.bo.it (R.D.C.); andrea.sambri2@unibo.it (A.S.)

**Keywords:** hip hemiarthroplasty, femoral neck fracture, postero-lateral approach, lateral approach, antero-lateral approach, anterior approach

## Abstract

*Background:* Femoral neck fractures are an epidemiologically significant issue with major effects on patients and health care systems, as they account for a large percentage of bone injuries in the elderly. Hip hemiarthroplasty is a common surgical procedure in the treatment of displaced femoral neck fractures. Several surgical approaches may be used to access the hip joint in case of femoral neck fractures, each with its own benefits and potential drawbacks, but none of them has consistently been found to be superior to the others. This article aims to systematically review and compare the different approaches in terms of the complication rate at the last follow-up. *Methods:* an in-depth search on PubMed/Scopus/Web of Science databases and a cross-referencing search was carried out concerning the articles comparing different approaches in hemiarthroplasty and reporting detailed data. *Results:* A total of 97,576 hips were included: 1030 treated with a direct anterior approach, 4131 with an anterolateral approach, 59,110 with a direct lateral approach, and 33,007 with a posterolateral approach. Comparing the different approaches, significant differences were found in both the overall complication rate and the rate of revision surgery performed (*p* < 0.05). In particular, the posterolateral approach showed a significantly higher complication rate than the lateral approach (8.4% vs. 3.2%, *p* < 0.001). Furthermore, the dislocation rate in the posterolateral group was significantly higher than in the other three groups considered (*p* < 0.026). However, the posterolateral group showed less blood loss than the anterolateral group (*p* < 0.001), a lower intraoperative fractures rate than the direct anterior group (*p* < 0.035), and shorter mean operative time than the direct lateral group (*p* < 0.018). *Conclusions:* The posterolateral approach showed a higher complication rate than direct lateral approach and a higher prosthetic dislocation rate than the other three types of surgical approaches. On the other hand, patients treated with posterolateral approach showed better outcomes in other parameters considered, such as mean operative time, mean blood loss and intraoperative fractures rate. The knowledge of the limitations of each approach and the most common associated complications can lead to choosing a surgical technique based on the patient’s individual risk.

## 1. Introduction

Fractures of the femoral neck are one of the most common bone injuries among the elderly, often caused by accidental trauma or bone fragility due to osteoporosis. This injury can cause intense pain and significantly limit a patient’s mobility, affecting their quality of life.

Hip hemiarthroplasty (HHA), also known as partial hip replacement is a common surgical procedure in the treatment of displaced femoral neck fractures. In this type of surgery, only the femoral head is replaced; the acetabulum is left intact and the joint is realigned to provide a smooth and stable surface for movement. HHA is considered a less invasive surgical technique than total hip arthroplasty and may be indicated especially for elderly patients or those with poor health conditions [1,2,3].

There are several surgical approaches that may be used to access the hip joint, each with its own benefits and potential drawbacks. The most appropriate approach for each patient depends on many factors, including general health status, the specific pathology, and the preference of the surgeon [3]. The direct lateral approach (DL) and posterolateral approach (PL) are the most commonly used according to the literature [4,5], but the anterolateral approach (AL) and the direct anterior approach (DA) have also been extensively described [6,7,8,9].

The complication rate of these approaches has been compared in several studies but none of them has consistently been found to be superior to the others. However, to our knowledge, there is no study in which all approaches have been analyzed simultaneously.

The aim of this systematic review of the literature is to offer an up-to-date overview of the evidence regarding hemiarthroplasty by comparing all the most used different approaches in terms of complication rate at last follow-up.

## 2. Materials and Methods

**Figure 1:**
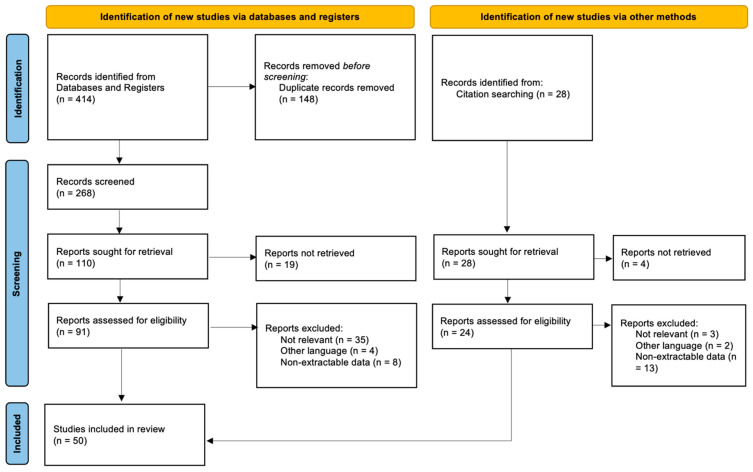
PRISMA flow diagram and the selection of studies.

This systematic review was conducted in accordance with the 2020 PRISMA guidelines (Preferred reporting items of systematic reviews) (Figure 1).

All studies (randomized controlled trials-RCT, prospective and retrospective comparative studies and case series) reporting on ‘hemiarthroplasty’ as treatment of femoral neck fractures were included. The diagnosis has been made based on clinical features and radiograph by the individual authors.

Studies reporting the results of femoral neck fractures treatment other than HHA (including THA and internal fixation techniques) were excluded. Studies reporting the results of HHA were included only if the results obtained in patients undergoing different surgical approaches were clearly distinguishable.

Only studies comparing two different surgical approaches were included. Only studies with a minimum follow-up of 6 months and a minimum of 5 patients treated with hemiarthroplasty available for analysis were considered for inclusion. Only studies in English were included. Case series reporting on a single technique were excluded. Biomechanical studies, cadaveric studies, in vitro studies, and animal model studies were also excluded.

Studies eligible for this systematic review have been identified, through an electronic systematic search with no restriction on date of publication, up to the end of February 2023, performed on PubMed (https://pubmed.ncbi.nlm.nih.gov/ (accessed on 28 February 2023)), Scopus (https://www.scopus.com (accessed on 28 February 2023)), and Web of Science (www.webofscience.com (accessed on 28 February 2023)) databases. Articles that were considered relevant by electronic search were retrieved in full-text, and a cross-referencing search of their bibliography was performed, to find further related articles. Reviews and meta-analyses were also analyzed, in order to broaden the search for studies that might have been missed through the electronic search. All duplicates were removed, and all the articles retrieved were analyzed.

After the first screening, records without eligibility criteria were excluded.

**Figure 2:**
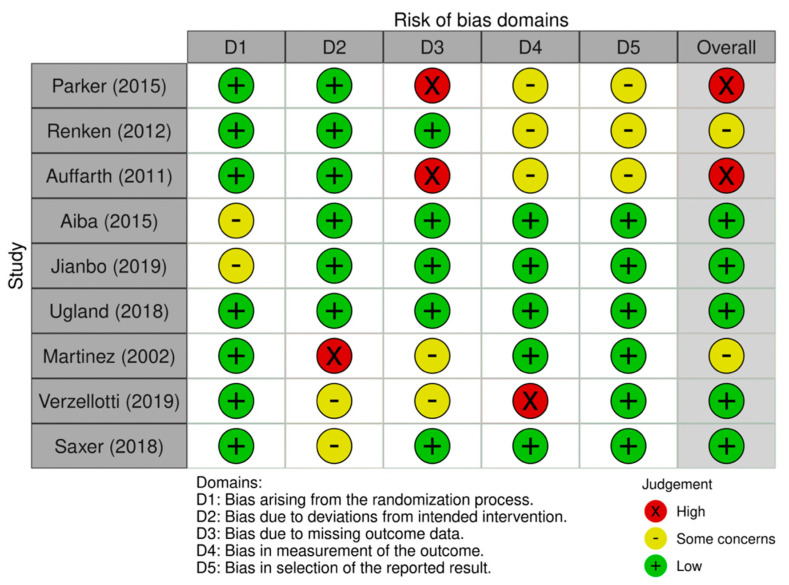
Cochrane risk-of-bias tool for randomized trials (RoB 2) [10,11,12,13,14,15,16,17,18].

**Figure 3:**
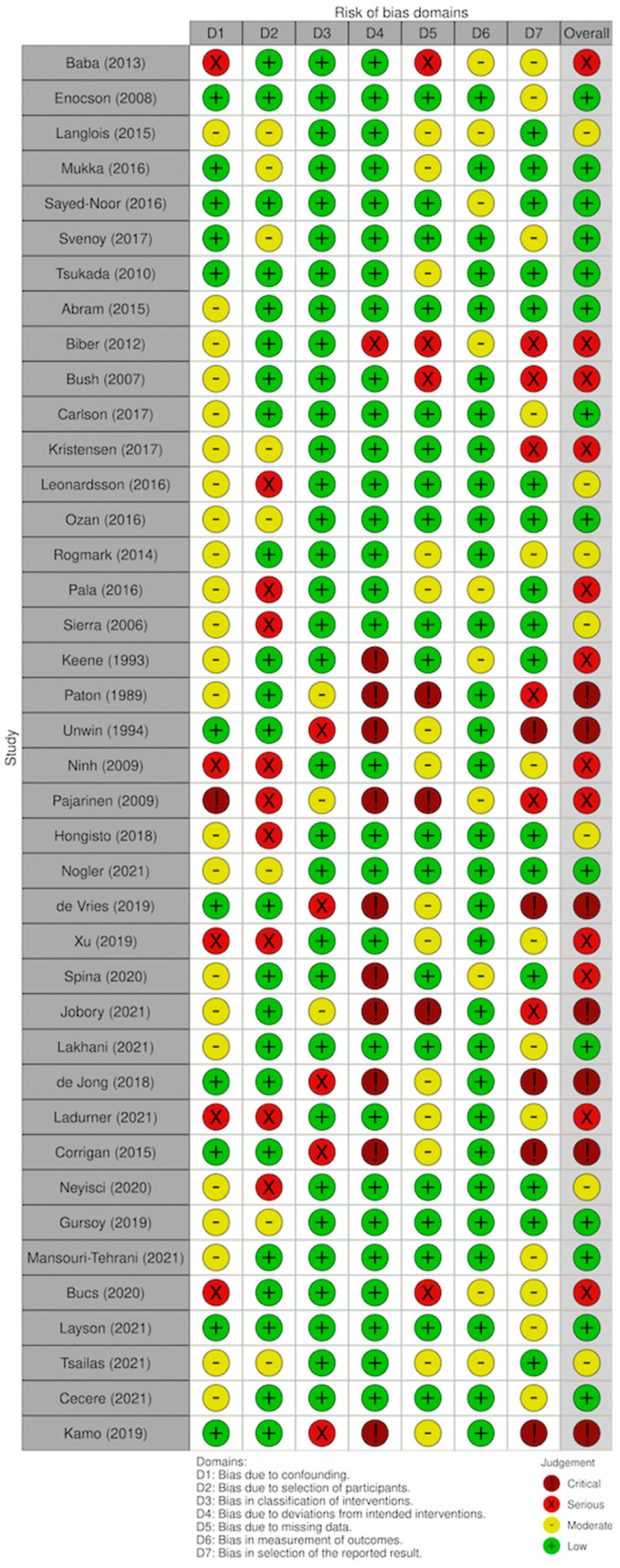
Cochrane risk-of-bias tool for non-randomized studies (ROBINS-I) [5,19,20,21,22,23,24,25,26,27,28,29,30,31,32,33,34,35,36,37,38,39,40,41,42,43,44,45,46,47,48,49,50,51,52,53,54,55,56,57,58].

Remnant studies were categorized by type, according to the Oxford Centre for Evidence-Based Medicine (OCEBM). To assess the quality of the articles, Cochrane risk-of-bias tool for randomized trials (RoB 2) (Figure 2) and Cochrane’s risk of bias tool for non-randomized studies (ROBINS-I) (Figure 3) were used. These tools assign a categorical value based on the risk of bias of each single aspect of each study and allow to obtain a summary value that quantifies its overall quality.

All the included studies were analyzed. The data extracted included mean age, mean follow-up, number of hips, mean operative time, mean estimated blood loss, number and type of peri-operative complications, number of revision surgeries, mean length of stay. Based on the type of surgical approach, four groups were formed: (1) direct lateral approach, (2) anterolateral approach, (3) direct anterior approach, (4) posterolateral approach. Functional outcomes were not reported in this review, due to the lack of these data in the vast majority of the included studies.

Studies with reported quantitative data were used for statistical analysis. Weighted means and standard deviations were calculated to summarize the values reported in the individual studies and to compare them. For quantitative variables, Shapiro–Wilk test was used to verify normal distribution. Levene test was used to assess the equality of variances. Chi-square statistics (Pearson’s chi-square, Yates’ chi-square, Fisher’s exact test, Fisher–Freeman–Halton test), ANOVA, or Kruskal–Wallis tests were used to assess associations and homogeneity among the groups, depending on the type of variables considered. The meta-analysis was conducted when at least 4 studies were available for comparison. Quantification of the extent of statistical heterogeneity across studies included in the meta-analysis employed the inconsistency statistic (I^2^ > 75% was considered as highly heterogeneity). Potential sources of heterogeneity by study-level and clinically relevant characteristics were explored using stratified analysis and meta-regression. Publication bias was assessed using Egger’s regression symmetry test. *p*-value < 0.05 was considered to be significant. All statistical analyses were performed with IBM SPSS v26.0 for MacOS (SPSS Inc., Chicago, IL, USA) and ProMeta 3 (Internovi, Cesena, Italy) softwares.

## 3. Results

A total of 268 studies were found through the electronic search and 3 studies were added follwing cross-referenced research on the bibliography of the examined full-text articles. After a preliminary analysis, a total of 50 studies were included in this systematic review [5,10,11,12,13,14,15,16,17,18,19,20,21,22,23,24,25,26,27,28,29,30,31,32,33,34,35,36,37,38,39,40,41,42,43,44,45,46,47,48,49,50,51,52,53,54,55,56,57,58] (Table 1).

The DL approach was compared to the AL approach in 3 studies [40,41,57], to the DA approach in 8 studies [10,18,39,41,44,46,57,58], and to the PL approach in 18 studies [5,11,20,23,25,26,27,28,34,36,37,40,41,42,45,51,52,57]. The AL approach was compared to the DA approach in five studies [13,41,48,49,57], and to the PL approach in nine studies [19,22,24,38,40,41,49,55,57]. The DA and the PL approach were compared in 10 studies [12,15,30,31,32,33,41,49,50,57].

Nine studies were randomized control trials [10,11,12,13,14,15,16,17,18], six were prospective comparative cohort studies [19,30,31,32,35,37], thirty-two were retrospective comparative cohort studies [20,21,22,23,24,26,27,28,29,33,34,36,38,39,40,41,42,43,44,46,47,48,49,50,51,52,53,54,55,56,57,58] and three were registry studies [5,25,45].

The overall quality of the series assessed (with Rob 2; Robins-I) was classified as high [12,14,15,16,18,22,24,33,35,36,37,39,41,46,51,52,54,56] or moderate [5,13,26,34,40,50,55] in most of the cases (Figure 2 and Figure 3). No significant differences were found between the different groups analyzed regarding the mean age and the mean follow-up time (Table 2).

A total of 97,278 hips were included: 1030 treated with the DA approach, 4131 treated with the AL approach, 59,110 treated with the DL approach, and 33,007 treated with the PL approach (Table 1). Mean age was comparable between the four groups (83.5 ± 3.0 in DA group; 82.2 ± 2.8 in AL group; 83.6 ± 3.0 in DL group; 83.8 ± 3.4 in PL group). The mean follow-up was 25.5 ± 11.3 months, comparable between the four groups.

**Figure 4:**
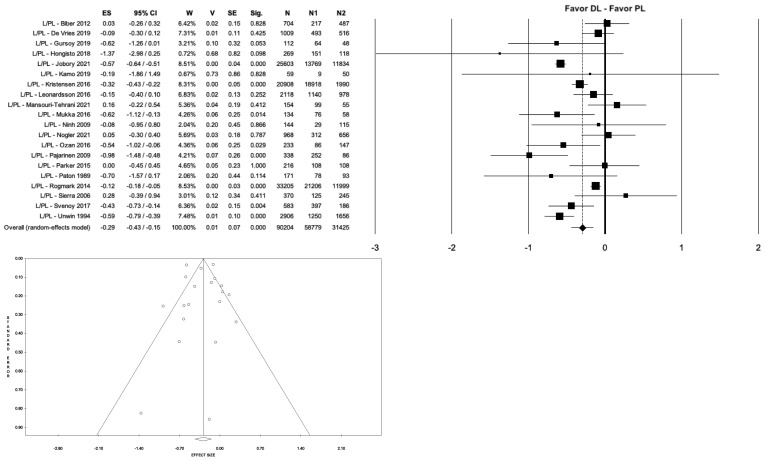
Forest plot and funnel plot of overall meta-analysis evaluating studies with data on overall complications in patients treated with direct lateral approach vs. posterolateral approach. The figure shows the highest estimated risk of complications in the posterolateral group. Abbreviations: ES, effect size; 95% CI, 95% confidence interval; W, weight; N, sample size [5,11,20,21,23,25,26,27,28,29,34,36,37,40,41,42,45,51,52,57].

Regarding the overall complication rate, significant differences were found between the different surgical approaches (*p* < 0.001). In particular, the PL approach showed a significantly higher complication rate than the DL approach (8.4% vs. 3.2%, *p* < 0.001, I^2^ = 86.13%) (Figure 4).

**Figure 5:**
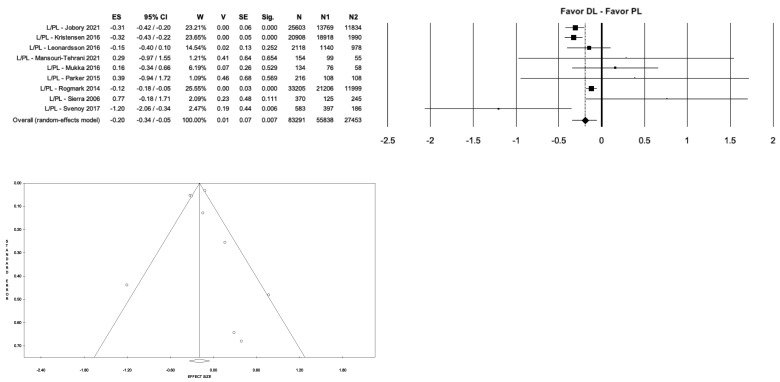
Forest plot and funnel plot of overall meta-analysis evaluating studies with data about revision surgeries in patients treated with direct lateral approach vs. posterolateral approach. The figure shows the highest estimated risk of revision surgeries in the posterolateral group. Abbreviations: ES, effect size; 95% CI, 95% confidence interval; W, weight; N, sample size [5,11,25,26,29,37,40,45,52].

The revision surgery rate also differed significantly between the individual surgical approaches (*p* < 0.001). In particular, compared to the DL approach, the PL group showed a significantly higher revision surgery rate (3.41% vs. 3.00%; *p* < 0.007, I^2^ = 71.52%), while the AL group showed a significantly lower revision surgery rate than the DL group (1.96% vs. 3.00%; *p* < 0.046; I^2^ ≈ 0%) (Figure 5).

**Figure 6:**
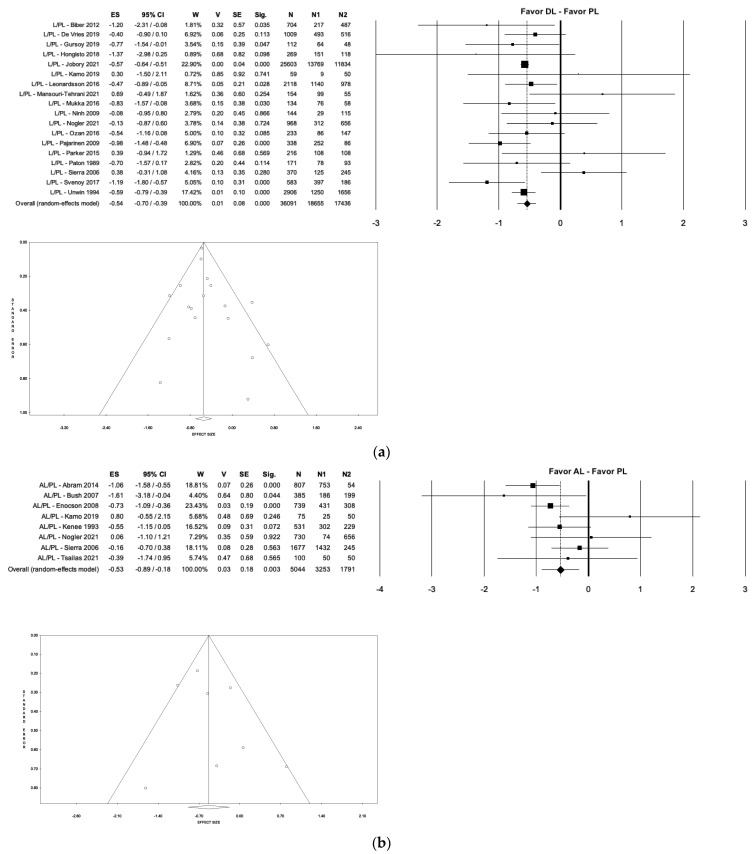

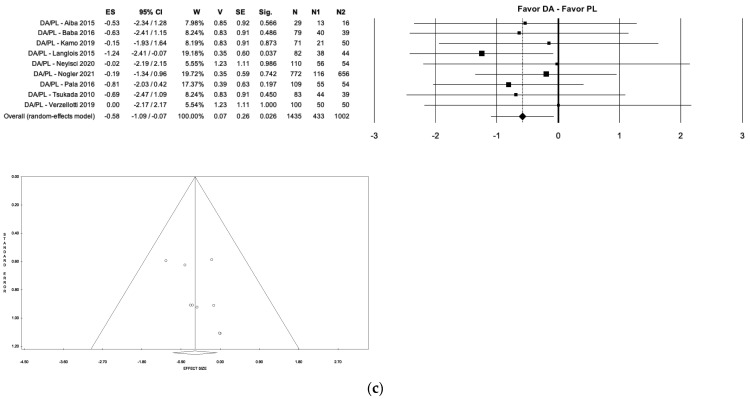
Forest plot and funnel plot of overall meta-analysis evaluating studies with data about dislocations in patients treated with direct lateral approach vs. posterolateral approach. (**a**) the figure shows the highest estimated risk of dislocations in the posterolateral group; anterolateral approach vs. posterolateral approach; (**b**) the figure shows the highest estimated risk of dislocations in the posterolateral group; direct anterior approach vs. posterolateral approach; (**c**) the figure shows the highest estimated risk of dislocations in the posterolateral group. Abbreviations: ES, effect size; 95% CI, 95% confidence interval; W, weight; N, sample size [11,12,15,19,20,21,22,23,24,26,27,28,29,30,31,32,33,34,36,37,38,40,41,42,45,50,51,52,55,57].

**Figure 7:**
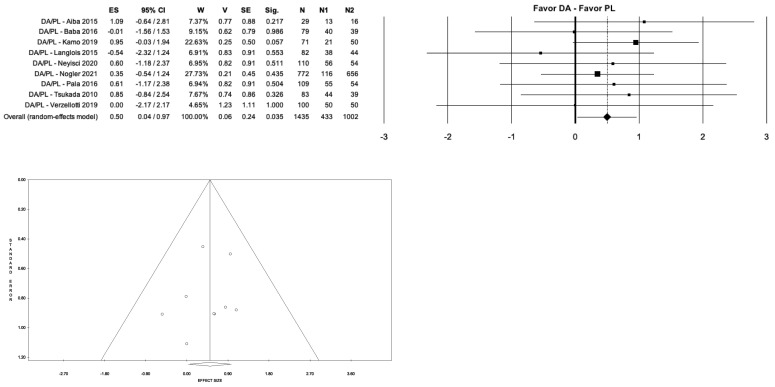
Forest plot and funnel plot of overall meta-analysis evaluating studies with data about intraoperative fractures in patients treated with direct anterior approach vs. posterolateral approach. The figure shows the highest estimated risk of dislocations in the direct anterior group. Abbreviations: ES, effect size; 95% CI, 95% confidence interval; W, weight; N, sample size [12,15,30,31,32,33,41,50,57].

**Figure 8:**
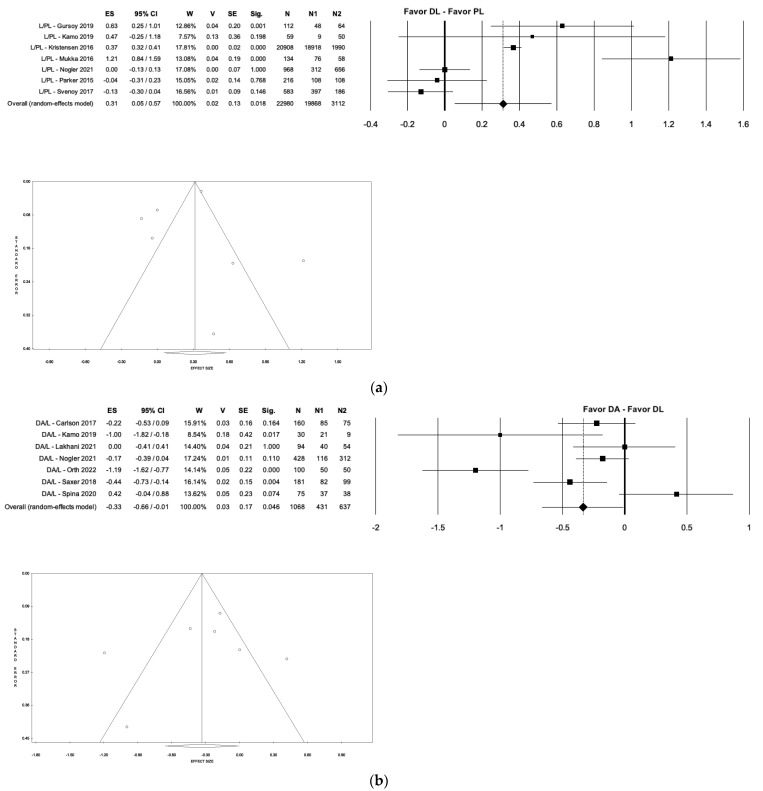
Forest plot and funnel plot of overall meta-analysis evaluating studies with data about mean operative time in patients treated with: direct lateral approach vs. posterolateral approach (**a**): the figure shows the shortest mean operative time in the posterolateral group; direct anterior approach vs. direct lateral approach (**b**): the figure shows the shortest mean operative time in the direct anterior group. Abbreviations: ES, effect size; 95% CI, 95% confidence interval; W, weight; N, sample size [11,18,25,29,37,39,41,44,46,51,57,58].

In this study, we compared the rate of each complication in the groups analyzed. It was found that the PL approach showed a significantly higher dislocation rate than the DA approach (5.10% vs. 0.68%; *p* < 0.035; I^2^ ≈ 0%), the AL group (5.10% vs. 1.62%; *p* < 0.001; I^2^ = 46.78%), and the DL group (5.10% vs. 1.54%; *p* < 0.018; I^2^ = 37.42%). No significant differences were found in dislocation rate when comparing the other three groups (DA, AL, DL) to each other (Figure 6). On the other hand, the PL group showed less mean blood loss than the AL group (359.63 mL vs. 449.5 mL; *p* < 0.001; I^2^ ≈ 0%), a lower intraoperative fractures rate than the DA group (0.13% vs. 1.26%; *p* < 0.035; I^2^ ≈ 0%) (Figure 7), and a shorter mean operative time than the DL group (69.38 min. vs. 78.04 min.; *p* < 0.018; I^2^ = 92.72%) (Figure 8a).

Furthermore, a significant difference was found concerning the mean length of stay between the AL and the DL group, with the AL group showing a greater length of stay (11.95 days vs. 8.56 days; *p* < 0.001; I^2^ ≈ 0%).

Other differences were observed between the DA group and the DL group, showing a lower mean operative time (74.23 min. vs. 78.04 min.; *p* < 0.046; I^2^ = 82.11%) (Figure 8b), but a higher mean blood loss (296.38 mL vs. 204.70 mL; *p* < 0.001; I^2^ ≈ 0%) in the DA group.

## 4. Discussion

This review was conducted with the aim of evaluating the evidence available in the literature regarding the differences between the four most common surgical approaches used for hemiarthroplasty surgery for femoral neck fractures. We found a large amount of data in the literature, but we decided to utilize only comparative studies, in order to conduct an “approach vs. approach” meta-analysis of good quality studies.

The distribution of patients according to the approach was not homogeneous, with a prevalence of cases treated with a PL and a DL approach. This is not to be understood as representative data of surgical practice, but is probably the consequence of the inclusion of registry studies, in which the included patients had been mainly treated with the PL or DL approach.

This study showed that none of the approaches analyzed was significantly worse overall in terms of total complications, in line with the findings of the study by Fullam et al. in 2018 [59]. The only cases in which significant differences were found has been when single “approach vs. approach” comparisons were conducted, also in line with the literature [11,23,31,32,37].

Significant differences in the overall complication rate were found only when comparing the DL approach with the PL approach, with the highest rate in the PL group. This finding contrasts with what was found by Tol et al. in 2021, with their systematic review, which showed no significant difference between the two groups [60]. This might be explained by the higher number of patients considered in the present review and the different quality of the studies included.

With regard to the rate of revision surgeries, this study appears to be in line with the literature. The re-operation rate in patients treated with the PL approach was significantly higher than in the DL group, similar to what emerged in the review by Van der Sijp et al. [61]. It deserves attention that the revision surgery rate in this review was found to be higher in the DL approach than in the AL approach. The reason might be related to the fact that the AL approach involves less muscle than the DL approach, despite the other similarities between these two approaches.

As far as single complications are concerned, significant differences emerged in the various comparisons with regard to the dislocation rate, intraoperative fractures, average blood loss, and mean operative time.

Data from this study suggest that the PL approach exposes the patient to an increased risk of dislocation than the other three surgical approaches analyzed. Tol et al. stated that the PL approach, compared with the DL approach, is associated with more dislocations, but patients have less walking problems and a lower risk of abductor insufficiency [60]. In addition, lateral patient positioning has the advantage of needing fewer operators for the procedure [61]. Similar results were found by Leonardsson et al., whose data showed that patients treated with the PL approach were affected by an increased risk of needing revision surgery due to dislocation, but had a better functional score at follow-up in terms of quality of life [26]. The review of the literature conducted by Van der Sijp et al. showed a higher risk of dislocation and reoperation in patients treated with the PL approach when compared with those treated with the DL and the DA approach [62]. It is interesting to note that the results of both this study and previous reviews regarding the higher dislocation rate of HHA performed with the PL approach differ from the results of total hip replacement (THA), according to the literature. In fact, with regard to THA, the dislocation rate appears to be comparable between the PL approach and the other ones. This is probably due both to the greater retention of the cup and to the possibility of positioning the acetabular components with different orientations, according to the chosen approach [63].

Another interesting finding from this review is the risk of intraoperative fractures, which was significantly lower in the PL approach than the DA approach, in agreement with the studies by Pala et al. and Langlois et al., who have previously compared complications related to these surgical approaches [31,32]. The higher rate of intraoperative fractures in the DA approach is probably due to the greater difficulty in correctly exposing the femur for its preparation, a maneuver which requires the application of greater force than in other approaches. These observations should be coupled with reporting the method of stem fixation used (cemented or uncemented), as this could be an important factor influencing fracture risk, regardless of the approach employed. However, as emphasized below in the section on study limitations, it was not possible to stratify data from many studies according to cementation. Nevertheless, considering the homogeneity of the mean age of patients for each approach (Table 2), always above 80 years, it might be reasonable to assume that the use of cemented stems was prominent in most studies, thus making it a non-determinant variable in influencing the results of this review on intraoperative fractures. This finding differs from what was found in the literature for THA surgery, for which, despite the absolute number of intraoperative fractures for the DA approach being higher, the difference does not appear to be significant when compared to the PL approach, as was found in the systematic review by Wang et al. [64]. This could be explained by advanced age and the greater number of comorbidities that can affect bone quality in patients treated with HA.

Few studies in the literature have compared the surgical time for the different approaches. The recent review by Kunkel et al. showed that there were no significant differences between the DA approach’s surgical time compared to the other approaches [65]. In this review, it was found that the PL approach has a significantly shorter surgical time than the DL approach. However, one aspect that should be considered is the time needed for patient positioning on the surgical bed, which is usually longer in the PL approach, as the patient lies on their side, compared to DL and DA approaches, in which the patient lies supine.

There are major limitations to this systematic review and meta-analysis. The studies included in this review have allowed a deep analysis for some comparisons between surgical approaches, but, on the other hand, have not allowed all possible comparisons, due to a lack of extensive data in the literature. Moreover, the limited number of randomized control studies available necessitated the inclusion of many non-randomized studies. Nevertheless, the rigorous methodological quality analysis performed has allowed us to identify several types of potential bias in the included studies. The analysis of functional outcomes was severely limited by cross-study variability, in the type of metrics used and patient follow-up duration. For these reasons, functional aspect was not included in this review. Moreover, analyzing the outcomes for every surgical approach by stratifying the cohorts according to the type of cup (unipolar versus bipolar) and the use of cement would have led to an excessive dispersion of data. In fact, these aspects were not able to be discriminated in most of the included studies. Therefore, a multivariate analysis that would allow the type of approach to be identified as an independent risk factor for specific outcomes was not performed.

## 5. Conclusions

In conclusion, there is no approach which appears worse overall, in terms of complications. This systematic literature review has showed that each approach has strengths and weaknesses. The posterolateral approach has the disadvantage of being characterized by a higher dislocation rate and a higher rate of complications than the DL approach. However, it has the advantage of having a shorter operative time, less blood loss, and the need for fewer operators. On the other hand, the DA approach carries the advantage of less blood loss, a shorter operating time, and a lower rate of dislocations compared to the PL approach. The disadvantage is the higher rate of intraoperative femoral fractures, which is why it would be less suitable for patients with greater risk of fracture due to poor bone quality. The DL approach shows a lower rate of complications and revisions than the PL, but has a longer operative time and greater blood loss. Knowledge of the limitations of each approach and the most common associated complications can lead to choosing a surgical technique based on the patient’s individual risk.

## Figures and Tables

**Table 1:** Data from the studies included in this review.

Study	Design	Approach	Total n. of Patiens	N. of Patients for Group	Mean Age (Years)	Mean FU (Months)	Mean OT (min)	Mean EBL (mL)	Local Peri-Operative Complications																Revision Surgeries		Mean LOS (Days)
									Dislocation		Stem Loosening		Periprosthetic Fracture		Deep Infection		Wound Dehiscence/Superficial Infection		Intraoperative Fracture		Others *		TOTAL				
									n.	%	n.	%	n.	%	n.	%	n.	%	n.	%	n.	%	n.	%	n.	%	N/A
Kenee (1993) [19]	PCCS	AL	531	302	81	12	56	251	5	1.7	0	0	0	0	6	2	18	6	6	2	4	1.3	39	12.9%	0	0	34
		PL		229	81		48	197	10	4.3	0	0	8	3.5	2	0.9	6	2.6	4	1.7	22	9.6	52	22.7%	0	0	33
Paton (1989) [20]	RCCS	DL	171	78	79.3	N/A	N/A	N/A	2	2.6	N/A	N/A	N/A	N/A	N/A	N/A	N/A	N/A	N/A	N/A	N/A	N/A	2	2.6%	N/A	N/A	N/A
		PL		93	79.3		N/A	N/A	8	8.6	N/A	N/A	N/A	N/A	N/A	N/A	N/A	N/A	N/A	N/A	N/A	N/A	8	8.6%	N/A	N/A	N/A
Unwin (1994) [21]	RCCS	DL	2906	1250	N/A	N/A	N/A	N/A	41	3.3	N/A	N/A	N/A	N/A	N/A	N/A	N/A	N/A	N/A	N/A	N/A	N/A	41	3.3%	N/A	N/A	N/A
		PL		1656		N/A	N/A	N/A	149	9	N/A	N/A	N/A	N/A	N/A	N/A	N/A	N/A	N/A	N/A	N/A	N/A	149	9.0%	N/A	N/A	N/A
Abram (2014) [22]	RCCS	AL	807	753	N/A	12	N/A	N/A	16	2.1	0	0	0	0	33	4.1	0	0	15	1.9	N/A	N/A	64	8.5%	33	4.1	26
		PL		54	N/A		N/A	N/A	7	13	0	0	0	0			0	0					7	13.0%			
Biber (2012) [23]	RCCS	PL	704	487	80.4	N/A	N/A	N/A	19	3.9	0	0	0	0	12	2.5	0	0	3	0.6	15	3	49	10.1%	N/A	N/A	N/A
		DL		217	80.3	N/A	N/A	N/A	1	0.5	0	0	0	0	7	3.2	0	0	1	0.5	14	6.4	23	10.6%	N/A	N/A	N/A
Enocson (2008) [24]	RCCS	AL	739	431	84	2.3	N/A	N/A	13	3	0	0	0	0	0	0	0	0	0	0	0	0	13	3.0%	13	1.8	N/A
		PL		308	85		N/A	N/A	32	10.4	0	0	0	0	0	0	0	0	0	0	0	0	32	10.4%			N/A
Kristensen (2016) [25]	RS	DL	20,908	18,918	83	36	76	N/A	N/A	N/A	N/A	N/A	N/A	N/A	N/A	N/A	N/A	N/A	N/A	N/A	N/A	N/A	0	0.0%	757	4	N/A
		PL		1990	83		67	N/A	N/A	N/A	N/A	N/A	N/A	N/A	N/A	N/A	N/A	N/A	N/A	N/A	N/A	N/A	0	0.0%	139	7	N/A
Leonardsson (2016) [26]	RCCS	DL	2118	1140	85	N/A	N/A	N/A	10	0.9	0	0	6	0.5	12	1.1	0	0	0	0	8	0.7	36	3.2%	36	3	N/A
		PL		978	85	N/A	N/A	N/A	20	2	0	0	4	0.4	13	1.3	0	0	0	0	3	0.3	40	4.1%	40	4	N/A
Ninh (2009) [27]	RCCS	PL	144	115	77.3	12	N/A	N/A	9	7.8	N/A	N/A	N/A	N/A	N/A	N/A	N/A	N/A	N/A	N/A	N/A	N/A	9	7.8%	N/A	N/A	N/A
		DL		29	77.3		N/A	N/A	2	6.9	N/A	N/A	N/A	N/A	N/A	N/A	N/A	N/A	N/A	N/A	N/A	N/A	2	6.9%	N/A	N/A	N/A
Pajarinen (2009) [28]	RCCS	PL	338	86	83.2	6	N/A	N/A	14	16.3	N/A	N/A	N/A	N/A	N/A	N/A	N/A	N/A	N/A	N/A	N/A	N/A	14	16.3%	N/A	N/A	N/A
		DL		252	83.2		N/A	N/A	8	3.2	N/A	N/A	N/A	N/A	N/A	N/A	N/A	N/A	N/A	N/A	N/A	N/A	8	3.2%	N/A	N/A	N/A
Parker (2015) [11]	RCT	DL	216	108	84.3	12	53.6	N/A	2	1.9	0	0	1	0.9	0	0	3	2.9	6	5.6	1	0.9	13	12.0%	2	1.9	20.3
		PL		108	83.6		54	N/A	1	0.9	0	0	4	3.8	2	1.9	2	1.9	2	1.9	2	1.9	13	12.0%	1	0.9	18.5
Rogmark (2014) [5]	RS	PL	33,205	11,999	84	32	N/A	N/A	443	1.3	13	0.04	154	0.5	424	1.3	N/A	N/A	N/A	N/A	130	0.4	1164	9.7%	477	4	N/A
		DL		21,206	84		N/A	N/A									N/A	N/A	N/A	N/A			0	0.0%	687	3.2	N/A
Svenøy (2017) [29]	RCCS	PL	583	186	83.2	12	69.2	N/A	15	8.1	N/A	N/A	N/A	N/A	N/A	N/A	12	6.5	3	1.6			30	16.1%	8	4.3	N/A
		DL		397	82.6		66.7	N/A	4	1	N/A	N/A	N/A	N/A	N/A	N/A	20	5	8	2			32	8.1%	2	0.5	N/A
Aiba (2015) [12]	RCT	DA	29	13	81.5	N/A	85.6	198.3	0	0	0	0	0	0	0	0	0	0	2	15	4	13.8	6	46.2%	0	0	N/A
		PA		16	78.6		61.8	146.7	0	0	0	0	0	0	0	0	0	0	0	0	3	10.3	3	18.8%	0	0	N/A
Auffarth (2011) [10]	RCT	DA	48	24	82.6	6	N/A	N/A	0	0	0	0	0	0	0	0	0	0	0	0	6	25	6	25.0%	1	4.2	N/A
		DL		24	83.7		N/A	N/A	0	0	0	0	0	0	1	4.2	0	0	1	4.2	2	8.3	4	16.7%	1	4.2	N/A
Renken (2012) [13]	RCT	DA	57	30	84	1.3	73.6	N/A	0	0	0	0	0	0	0	0	1	3.3	0	0	1	3.3	2	6.7%	0	0	N/A
		AL		27	87.5		64.8	N/A	0	0	0	0	0	0	1	3.7	0	0	0	0	2	7.4	3	11.1%	0	0	N/A
Baba (2013) [30]	PCCS	DA	79	40	76.7	36	65.3	121	0	0	0	0	0	0	0	0	0	0	1	2.5	0	0	1	2.5%	0	0	29.9
		PL		39	74.9		76.7	146	1	2.6	0	0	0	0	0	0	0	0	1	2.6	0	0	2	5.1%	0	0	29.3
Langlois (2015) [31]	PCCS	DA	82	38	86	22	65	N/A	1	2.6	0	0	0	0	0	0	0	0	0	0	2	5.3	3	7.9%	1	2.6	N/A
		PL		44	75		54	N/A	9	20.5	0	0	0	0	1	2.3	0	0	1	2.3	1	2.3	12	27.3%	1	2.3	N/A
Pala (2016) [32]	PCCS	DA	109	55	89	24	47	289	1	1.8	0	0	0	0	0	0	0	0	1	1.8	3	5.5	5	9.1%	N/A	N/A	12
		PL		54	87.6		57	213	4	7.4	0	0	1	1.8	0	0	0	0	0	0	1	1.8	6	11.1%	N/A	N/A	14
Tsukada (2010) [33]	RCCS	DA	83	44	80.4	12	75.1	370.1	0	0	0	0	0	0	0	0	0	0	2	4.5	1	2.3	3	6.8%	0	0	35.4
		PL		39	81.9		79.3	230	1	2.6	0	0	0	0	0	0	0	0	0	0	0	0	1	2.6%	0	0	36.1
Hongisto (2018) [34]	RCCS	DL	269	151	82.9	12	N/A	N/A	0	0	N/A	N/A	N/A	N/A	N/A	N/A	N/A	N/A	N/A	N/A	N/A	N/A	0	0.0%	N/A	N/A	N/A
		PL		118	82.5		N/A	N/A	4	3.4	N/A	N/A	N/A	N/A	N/A	N/A	N/A	N/A	N/A	N/A	N/A	N/A	4	3.4%	N/A	N/A	N/A
Sayed-Noor (2016) [35]	PCCS	DL	48	24	83.4	12	N/A	N/A	N/A	N/A	N/A	N/A	N/A	N/A	N/A	N/A	N/A	N/A	N/A	N/A	N/A	N/A	0	0.0%	N/A	N/A	N/A
		PL		24	82.7		N/A	N/A	N/A	N/A	N/A	N/A	N/A	N/A	N/A	N/A	N/A	N/A	N/A	N/A	N/A	N/A	0	0.0%	N/A	N/A	N/A
Ozan (2016) [36]	RCCS	DL	233	86	78.3	17.1	N/A	N/A	4	4.6	0	0	0	0	3	3.4	0	0	0	0	N/A	N/A	7	8.1%	N/A	N/A	N/A
		PL		147	78.7		N/A	N/A	17	11.5	0	0	0	0	11	7.4	0	0	0	0			28	19.0%	N/A	N/A	N/A
Mukka (2016) [37]	PCCS	DL	185	76	83.5	12	90	254	3	3.9	0	0	1	1.3	5	6.6	0	0	0	0	0	0	9	11.8%	15	19.7	N/A
		PL		58	85.5		66	239	9	15.5	0	0	0	0	5	8.6	2	3.4	0	0	1	1.7	17	29.3%	9	15.5	N/A
Bush (2007) [38]	RCCS	AL	385	186	80.5	6	N/A	N/A	0	0	0	0	0	0	0	0	0	0	0	0	0	0	0	0.0%	N/A	N/A	7.3
		PL		199	79.2		N/A	N/A	9	4.5	1	0.5	0	0	0	0	0	0	0	0	1	0.5	11	5.5%	N/A	N/A	6.4
Carlson (2017) [39]	RCCS	DA	160	85	82.7	6	42.9	N/A	2	2.4	0	0	3	3.5	1	1.2	0	0	0	0	2	2.4	8	9.4%	4	4.7	6.2
		DL		75	82.9		N/A	N/A	0	0	0	0	3	4	2	2.7	0	0	0	0	3	4	8	10.7%	5	6.7	8.9
Sierra (2006) [40]	RCCS	AL	1802	1432	N/A	N/A	N/A	N/A	22	1.5	N/A	N/A	3	0.2	N/A	N/A	N/A	N/A	N/A	N/A	N/A	N/A	25	1.7%	15	1	N/A
		PL		245	N/A	N/A	N/A	N/A	5	2	N/A	N/A	1	0.4	N/A	N/A	N/A	N/A	N/A	N/A	N/A	N/A	6	2.4%	2	0.8	N/A
		DL		125	N/A	N/A	N/A	N/A	5	4	N/A	N/A	0	0	N/A	N/A	N/A	N/A	N/A	N/A	N/A	N/A	5	4.0%	4	3.2	N/A
Nogler (2021) [41]	RCCS	PL	1158	656	89.1	N/A	N/A	N/A	8	1.2	N/A	N/A	15	2.28	N/A	N/A	N/A	N/A	6	0.9	N/A	N/A	29	4.4%	N/A	N/A	4.2
		DL		312	86.7	N/A	N/A	N/A	3	0.96	N/A	N/A	8	2.56	N/A	N/A	N/A	N/A	4	1.28	N/A	N/A	15	4.8%	N/A	N/A	4.8
		DA		116	85	N/A	N/A	N/A	1	0.86	N/A	N/A	1	0.86	N/A	N/A	N/A	N/A	2	1.7	N/A	N/A	4	3.4%	N/A	N/A	2.3
		AL		74	84.7	N/A	N/A	N/A	1	1.35	N/A	N/A	1	1.35	N/A	N/A	N/A	N/A	2	2.7	N/A	N/A	4	5.4%	N/A	N/A	2.8
de Vries (2019) [42]	RCCS	DL	1009	493	87	N/A	N/A	N/A	7	1.4	N/A	N/A	14	2.8	23	4.5	11	2.2	N/A	N/A	N/A	N/A	55	11.2%	N/A	N/A	7
		PL		516	86	N/A	N/A	N/A	15	2.9	N/A	N/A	12	2.3	23	4.7	16	3.1	N/A	N/A	N/A	N/A	66	12.8%	N/A	N/A	7
Spina (2020) [44]	RCCS	DA	75	37	87.6	12	87.7	N/A	1	2.7	0	0	0	0	0	0	0	0	0	0	N/A	N/A	1	2.7%	N/A	N/A	N/A
		DL		38	87		82	N/A	2	5.3	0	0	0	0	1	2.6	0	0	0	0	N/A	N/A	3	7.9%	N/A	N/A	N/A
Jobory (2021) [45]	RS	DL	25,603	13,769	N/A	12	N/A	N/A	366	2.7	N/A	N/A	N/A	N/A	N/A	N/A	N/A	N/A	N/A	N/A	N/A	N/A	366	2.7%	162	1.2	N/A
		PL		11,834	N/A		N/A	N/A	850	7.2	N/A	N/A	N/A	N/A	N/A	N/A	N/A	N/A	N/A	N/A	N/A	N/A	850	7.2%	241	2	N/A
Lakhani (2021) [46]	RCCS	DA	94	40	85.4	19.2	90	N/A	1	2.5	0	0	0	0	2	5	0	0	0	0	2	5	5	12.5%	2	5	8
		DL		54	85.8		90	N/A	2	3.7	0	0	0	0	4	7.4	0	0	2	3.7	2	3.7	10	18.5%	5	9.26	9
Verzellotti (2019) [15]	RCT	DA	100	50	85.3	6	72.6	N/A	0	0	0	0	0	0	0	0	0	0	0	0	5	10	5	10.0%	0	0	N/A
		PL		50	85		64.1	N/A	0	0	0	0	0	0	0	0	0	0	0	0	6	12	6	12.0%	0	0	N/A
Ugland (2018) [16]	RCT	AL	150	75	81.4	12	N/A	N/A	N/A	N/A	N/A	N/A	N/A	N/A	N/A	N/A	N/A	N/A	N/A	N/A	N/A	N/A	0	0.0%	N/A	N/A	N/A
		DL		75	81.3		N/A	N/A	N/A	N/A	N/A	N/A	N/A	N/A	N/A	N/A	N/A	N/A	N/A	N/A	N/A	N/A	0	0.0%	N/A	N/A	N/A
Ladurner (2021) [48]	RCCS	DA	237	79	85.5	N/A	72.5	285.5	0	0	0	0	0	0	1	1.3	0	0	0	0	2	2.5	3	3.8%	2	2.5	8.3
		AL		158	86		89.5	287	1	0.6	0	0	0	0	2	1.3	0	0	0	0	8	5.1	11	7.0%	5	3.2	8.4
Corrigan (2015) [49]	RCCS	DA	82	26	78.5	N/A	N/A	N/A	N/A	N/A	N/A	N/A	N/A	N/A	N/A	N/A	N/A	N/A	N/A	N/A	5	19	5	19.2%	N/A	N/A	N/A
		AL		32	77.3		N/A	N/A	N/A	N/A	N/A	N/A	N/A	N/A	N/A	N/A	N/A	N/A	N/A	N/A	11	34	11	34.4%	N/A	N/A	N/A
		PL		24	81.7		N/A	N/A	N/A	N/A	N/A	N/A	N/A	N/A	N/A	N/A	N/A	N/A	N/A	N/A	6	25	6	25.0%	N/A	N/A	N/A
Neyisci (2020) [50]	RCCS	PL	110	54	83	15.5	110	N/A	0	0	0	0	0	0	0	0	0	0	0	0	0	0	0	0.0%	0	0	11.3
		DA		56	82		90	N/A	0	0	0	0	0	0	0	0	0	0	1	1.8	3	5.4	4	7.1%	0	0	8.2
Gursoy (2019) [51]	RCCS	PL	112	48	86.5	42	66.6	N/A	8	16.7	0	0	0	0	2	4.2	0	0	0	0	N/A	N/A	10	20.8%	N/A	N/A	N/A
		DL		64	87.1		60	N/A	3	4.7	0	0	0	0	2	3.1	0	0	0	0	N/A	N/A	5	7.8%	N/A	N/A	N/A
Mansouri-Tehrani (2021) [52]	RCCS	DL	154	99	78	36.5	N/A	N/A	6	6.1	0	0	0	0	4	4.04	0	0	0	0	29	29.3	39	39.4%	3	3.03	N/A
		PL		55	75.4		N/A	N/A	1	1.81	0	0	0	0	2	3.63	0	0	0	0	15	27.3	18	32.7%	1	1.81	N/A
Bucs (2020) [53]	RCCS	DA	94	51	79.4	4	52.3	738.23	N/A	N/A	N/A	N/A	N/A	N/A	N/A	N/A	N/A	N/A	N/A	N/A	N/A	N/A	0	0.0%	N/A	N/A	1.4
		AL		43	79.3		53.7	810.47	N/A	N/A	N/A	N/A	N/A	N/A	N/A	N/A	N/A	N/A	N/A	N/A	N/A	N/A	0	0.0%	N/A	N/A	3.1
Layson (2021) [54]	RCCS	DA	173	93	81.6	N/A	95.1	N/A	N/A	N/A	N/A	N/A	N/A	N/A	N/A	N/A	N/A	N/A	N/A	N/A	N/A	N/A	0	0.0%	N/A	N/A	N/A
		AL		80	79.1		74.8	N/A	N/A	N/A	N/A	N/A	N/A	N/A	N/A	N/A	N/A	N/A	N/A	N/A	N/A	N/A	0	0.0%	N/A	N/A	N/A
Saxer (2018) [18]	RCT	DL	181	99	84	12	100.1	N/A	0	0	0	0	0	0	0	0	5	5.1	0	0	46	46.5	51	51.5%	N/A	N/A	N/A
		DA		82	84.4		96.3	N/A	0	0	0	0	0	0	7	8.5	0	0	0	0	0	0	7	8.5%	N/A	N/A	N/A
Tsailas (2021) [55]	RCCS	AL	100	50	80.9	47	75	N/A	1	2	0	0	0	0	0	0	0	0	2	4	2	4	5	10.0%	N/A	N/A	N/A
		PL		50	82.3		67.5	N/A	2	4	0	0	0	0	0	0	0	0	2	4	5	10	9	18.0%	N/A	N/A	N/A
Kamo (2019) [57]	RCCS	AL	194	25	82.2	10	80	N/A	2	8	0	0	6	3.1	0	0	0	0	4	16	6	3.1	18	72.0%	N/A	N/A	N/A
		DA		21	83		63	N/A	0	0	0	0			0	0	0	0	4	19			4	19.0%	N/A	N/A	N/A
		DL		9	87.1		82	N/A	0	0	0	0			0	0	0	0	0	0			0	0.0%	N/A	N/A	N/A
		PL		50	83.6		72	N/A	1	2	0	0			0	0	0	0	2	4			3	6.0%	N/A	N/A	N/A
Orth (2022) [58]	RCCS	DA	100	50	82.5	12	86.9	72.5	0	0	0	0	0	0	1	2	0	0	0	0	N/A	N/A	1	2.0%	N/A	N/A	13.3
		DL		50	79.9		90.7	155.4	1	2	0	0	0	0	0	0	0	0	3	6	N/A	N/A	4	8.0%	N/A	N/A	13.1

* Deep vein thrombosis, pulmonary embolism, hematoma, seroma, sepsis, cardiovascular accident, acetabular erosion, nerve palsy, heterotopic ossification. Abbreviations: FU, follow-up; OT, operative time; EBL, estimated blood loss; LOS, length of stay; PCCS, prospective comparative cohort study; RCCS, retrospective comparative cohort study; RS, registry study; RCT, randomized controlled trial; DA, direct anterior approach; AL, anterolateral approach; DL, direct lateral approach; PL, posterolateral approach; N/A, not available.

**Table 2:** Summarized data from the included studies of this review.

	DA	AL	DL	PL	Total
Studies (n.)	20	16	27	41	50
N. of patients	1030	4131	59,110	33,007	97,576
Mean age (yrs)	83.5	82.2	83.6	83.8	83.4
Mean follow-up (months)	13.1	9.8	28.0	22.7	25.5
Complications (%)	79 (7.7)	258 (6.2)	1901 (3.2)	2762 (8.4)	3773 (3.9)
Revision surgery (%)	10 (2.0)	70 (2.9)	1677 (3.0)	965 (3.4)	2678 (3.0)

Abbreviations: DA, direct anterior approach; AL, anterolateral approach; DL, direct lateral approach; PL, posterolateral approach.

## Data Availability

Data are available on current Literature.
